# Lung Cancer Prevalence in Virginia: A Spatial Zipcode-Level Analysis via INLA

**DOI:** 10.3390/curroncol31030084

**Published:** 2024-02-20

**Authors:** Indranil Sahoo, Jinlei Zhao, Xiaoyan Deng, Myles Gordon Cockburn, Kathy Tossas, Robert Winn, Dipankar Bandyopadhyay

**Affiliations:** 1 Department of Statistical Sciences and Operations Research, Virginia Commonwealth University, Richmond, VA 23284, USA; sahooi@vcu.edu; 2 Massey Cancer Center, Virginia Commonwealth University, Richmond, VA 23284, USA; jzhao4@vcu.edu (J.Z.); katherine.tossas@vcuhealth.org (K.T.); robert.winn@vcuhealth.org (R.W.); 3 Department of Biostatistics, Virginia Commonwealth University, Richmond, VA 23284, USA; xdeng@vcu.edu; 4 Norris Comprehensive Cancer Center, Kerck School of Medicine, University of Southern California, Los Angeles, CA 90089, USA; cockburn@usc.edu

**Keywords:** discrete response, INLA, lung cancer, missing covariate imputation, spatial data, zip code-level analysis

## Abstract

Background: Examining lung cancer (LC) cases in Virginia (VA) is essential due to its significant public health implications. By studying demographic, environmental, and socioeconomic variables, this paper aims to provide insights into the underlying drivers of LC prevalence in the state adjusted for spatial associations at the zipcode level. Methods: We model the available VA zipcode-level LC counts via (spatial) Poisson and negative binomial regression models, taking into account missing covariate data, zipcode-level spatial association and allow for overdispersion. Under latent Gaussian Markov Random Field (GMRF) assumptions, our Bayesian hierarchical model powered by Integrated Nested Laplace Approximation (INLA) considers simultaneous (spatial) imputation of all missing covariates through elegant prediction. The spatial random effect across zip codes follows a Conditional Autoregressive (CAR) prior. Results: Zip codes with elevated smoking indices demonstrated a corresponding increase in LC counts, underscoring the well-established connection between smoking and LC. Additionally, we observed a notable correlation between higher Social Deprivation Index (SDI) scores and increased LC counts, aligning with the prevalent pattern of heightened LC prevalence in regions characterized by lower income and education levels. On the demographic level, our findings indicated higher LC counts in zip codes with larger White and Black populations (with Whites having higher prevalence than Blacks), lower counts in zip codes with higher Hispanic populations (compared to non-Hispanics), and higher prevalence among women compared to men. Furthermore, zip codes with a larger population of elderly people (age ≥ 65 years) exhibited higher LC prevalence, consistent with established national patterns. Conclusions: This comprehensive analysis contributes to our understanding of the complex interplay of demographic and socioeconomic factors influencing LC disparities in VA at the zip code level, providing valuable information for targeted public health interventions and resource allocation. Implementation code is available at GitHub.

## 1. Introduction

According to the American Cancer Society (https://www.cancer.org/research/cancer-facts-statistics/all-cancer-facts-figures/2023-cancer-facts-figures.html; accessed on 17 January 2024), lung cancer (LC) is the second most common type of cancer in both men and women when not accounting for skin cancer. The American Cancer Society has estimated a total of 238,340 new cases of LC in the United States in 2023, of which 117,550 were projected to be in men and 120,790 in women [1]. LC is the leading cause of cancer-related deaths in both genders [2], accounting for about 1 in 5 of all cancer deaths and accounting for more deaths than breast, prostate, and colon cancers combined. Similar trends are seen in Virginia (VA), where LC is the third most prevalent type of cancer, after breast cancer (in females) and prostate cancer (in men). As of 2023, the rate of new age-adjusted LC incidences per 100,000 individuals in VA is 52.4, significantly lower than the national rate of 54.6, placing it in the average tier (16th) among all states. Consistent with the national trend, LC in VA exhibits the highest mortality rate compared to other types of cancer [1].

Over the past few decades, a large body of research has focused on determining factors which are causally associated with LC, and studies have been implemented to identify determinants of susceptibility to these factors. Smoking has been identified as the most prevalent cause of LC in both men and women [3,4,5,6,7]. A state-wide study in California attributed declining LC mortality rates in the state to declines in smoking due to the California Tobacco Control Program [8]. Overall, smoking accounts for at least 30% of all cancer deaths and 87% of LC deaths [9]. State-specific trends in LC prevalence and smoking between 1999 and 2008 have been examined in [10]. Other causes of LC include positive family history of LC [11], (red) meat-rich diet [12], (smoking-adjusted) alcohol consumption [13,14], low socioeconomic status [15], occupational risk factors such as asbestos [16], arsenic [17], and radon [18], and other environmental factors such as passive smoking and air pollution [3].

Over the last 30 years, a plethora of statistical models have been used to project LC prevalence and mortality rates. A majority of these models are common forms of generalized linear models (GLM), where the number of cases (say, deaths) or the logarithm thereof is modeled as a linear or nonlinear function of the explanatory factors via Poisson or negative binomial (NB) distributions, with the logarithm of the population size as an offset adjustment (see Yu et al. [19] for a comprehensive literature review). Interestingly, their review suggests that a vast majority (about 87%) of studies did not incorporate data on smoking in the population. In recent times, the increased availability of georeferenced health information and population data have warranted investigations of spatial and spatiotemporal variations in LC prevalence. For example, the geographic variation of LC prevalence in Florida between 2000 and 2011 was studied in [20] using a spatial filtering technique, and the spatial variation of LC counts in New Jersey was studied in [21] using SaTScan software. In New Hampshire, a geocomputational clustering process based on kernel density estimation was implemented to create high-resolution disease incidence maps [22]. More recently, in Kentucky, a state with high rates of smoking and obesity, multinomial and discrete Poisson spatiotemporal scan statistics adjusted for age, gender, and race were used to investigate the spatial and temporal distribution of LC histological types [23]. Finally, a Bayesian geostatistical model for analyzing LC data in Ohio was demonstrated in [24,25].

In this paper, we focus on analyzing LC counts in VA obtained at the zip code level during the years 2014–2018. The central research question addressed in our paper involves investigating the impact of covariates on aggregated LC counts during these five years while accounting for overdispersion, spatial association at the zip code level, and potential missingness in covariate information. We explore the use of Poisson and NB spatial regression models to predict LC counts, thereby allowing for overdispersion in the response. The spatial correlation across zip codes is modeled using the popular Conditional Autoregressive (CAR) model [26,27]. We fit the model using the Integrated Nested Laplace Approximation (INLA) [28] framework for (approximate) Bayesian inference, which is a computationally effective alternative to usual Bayesian predictive inference implemented via Markov chain Monte Carlo (MCMC)-based methods. One salient feature of our data is that covariates relevant to LC, such as prevalence of smoking and binge drinking, often encounter missing values. Earlier studies on LC have considered either entirely removing covariates with missing values or employing imputation techniques [29]. However, (entire) covariate removal may lead to imprecise and misleading conclusions, and these available imputing techniques were not constructed with accommodation of possible spatial associations, as in our case. To circumvent this issue, we implement a recently proposed simultaneous spatial imputation technique [30]. We consider covariates with missing values as latent Gaussian Markov Random Fields (GMRFs) [31] and utilize Bayesian hierarchical modeling within the INLA framework. The latent effects required to impute the missing covariates were implemented using the MIINLA package in R (available from Github repository at https://github.com/becarioprecario/MIINLA; accessed on 17 January 2024). In addition to this novel application of integrating missing covariate imputations within the INLA framework, we provide reproducible and comprehensive implementation code accompanied by instructions at https://github.com/indranil09/VA_LungCancer_INLA; accessed on 17 January 2024. This inclusion facilitates the implementation and generalization of our analytical tool to other databases, particularly those pertaining to cancer incidence and prevalence, with similar data characteristics.

## 2. Materials and Methods

### 2.1. Ethics Statement

This study was approved by the Institutional Review Board of the Virginia Commonwealth University with protocol number HM20022764.

### 2.2. Data Description

**Figure 1:**
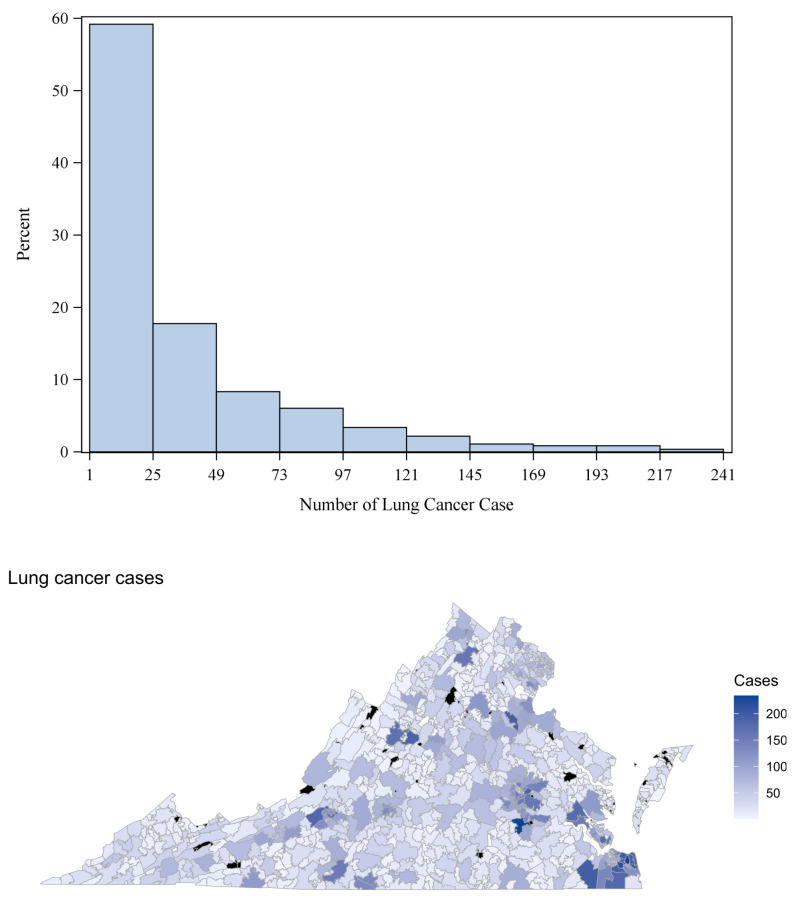
Top Panel: histogram of LC counts; the x-axis of the histogram starts at 1, indicating that there are no zeroes in the response. Bottom Panel: zip code-level spatial map of LC counts in VA; zip codes with missing (NA) responses are colored black.

The data used in this zip code-level analysis of LC counts in VA focuses on adult population (age ≥ 18). Of the 896 zip codes in VA, five zip codes (22035, 22185, 22214, 23250, 24316), including zip codes belonging to airport areas, were excluded due to the unavailability of any data. Additionally, zip code 23440 (the zip code for Tangier island, VA) was removed as it did not have credible spatial adjacency, making it unsuitable for spatial analysis. The numbers of LC cases in each zip code area during the years 2014–2018 were obtained from the VA Cancer Registry (https://www.vdh.virginia.gov/virginia-cancer-registry; accessed on 17 January 2024), which is maintained by the VA Department of Health. Among the 890 zip code data records included in our analysis, 62 (approximately 7%) had missing LC counts. Based on the 828 observed counts, the average number of LC cases in VA over the study period was 33.22, with a variance of 1746.15 (SD = 41.8). This is indicative of overdispersion, as the variance of the data exceeds the mean. To measure the extent of spatial autocorrelation in the response, the global Moran’s I statistic [32] was calculated. The estimated Moran’s I was 0.263 (*p*-value = 0.0009 < 0.01), indicating moderate spatial correlation. Figure 1 presents the histogram of LC counts along with its spatial distribution at the zip code level. The x-axis of the histogram starts at 1, indicating that there are no 0 (zero) values for the response in our dataset. The spatial map shows highest counts of LC in the southeastern counties of VA Beach, Suffolk, and Chesapeake, along with Petersburg, the City of Richmond, and their neighboring counties in central VA. We see somewhat high LC cases in Fairfax and Arlington counties of northern VA as well. Table 1 lists the top 15 zipcodes in VA with the highest LC counts over the study period (2014–2018).

Based on the important factors identified in previous studies, the covariates in our study included demographic information, social deprivation index (SDI), prevalence of smoking, binge drinking, and obesity in the population, average daily PM2.5 concentration, and percentage of population living below the poverty level. Brief descriptions of these covariates are provided below.

#### 2.2.1. Demographics

The demographic variables included in the study were Gender, Race, Ethnicity, and Age. In particular, we considered the variable ‘percentage of males’ in our study, as it can be used to infer the likelihood of males contracting LC compared to females. Similarly, we considered the variables ‘percentage of Blacks’, ‘percentage of Whites’ and ‘percentage of Hispanics’ to quantify the impact of Race and Ethnicity on LC counts. The impact of race, specifically Black or White, on the prevalence of LC was measured against the category ‘Other’, which includes Asian, Native Hawaiians and Other Pacific Islanders (NHPI), American Indian and Alaska Natives, some other race alone, and two or more races. Additionally, the effect of age was incorporated into the model through the variable ‘percentage of population with age ≥ 65’. Data on these four variables were obtained from the American Community Survey (ACS) in US Census (https://data.census.gov/advanced; accessed on 17 January 2024; see tables S0101, B01001A, B01001B, B01001I).

#### 2.2.2. Prevalence of Binge Drinking, Smoking, and Obesity

Data on prevalence of binge drinking, smoking, and obesity were obtained from the model-based zip code-level estimates for the PLACES (Population Level Analysis and Community Estimates) project 2020 (www.cdc.gov/places; accessed on 17 January 2024). A joint collaboration between the Centers for Disease Control and Prevention (CDC), the Robert Wood Johnson Foundation (RWJF), and the CDC Foundation (CDCF), the PLACES project is an extension of the 500 Cities Project, providing estimates for chronic disease risk factors, health outcomes, and clinical preventive services use by all counties, places (incorporated and census-designated places), census tracts, and zip codes across the United States. Data sources used to generate these model-based estimates include the Behavioral Risk Factor Surveillance System (BRFSS, www.cdc.gov/BRFSS; accessed on 17 January 2024) 2017 and 2018 data, Census Bureau 2010 population estimates, and American Community Survey (ACS) 2013–2017 and 2014–2018 estimates. For this study, we extracted data on the relevant variables from the Chronic Disease and Health Promotion data portal (https://chronicdata.cdc.gov/500-Cities-Places/PLACES-ZCTA-Data-GIS-Friendly-Format-2020-release/bdsk-unrd; accessed on 17 January 2024).

#### 2.2.3. Percent Population below Poverty

Data on the percentage of the population living below poverty in VA were obtained from the ACS in the US Census (https://data.census.gov/advanced; accessed on 17 January 2024; tables S1701).

#### 2.2.4. Social Deprivation Index (SDI)

The Social Deprivation Index (SDI) [33,34] is a composite measure of an area’s level of deprivation based on seven demographic characteristics collected in the ACS and used to quantify the socioeconomic variation in health outcomes. The seven characteristics are percent living in poverty, percent with less than 12 years of education, percent single-parent households, percent living in rented housing units, percent living in overcrowded housing units, percent of households without a car, and percent unemployed adults under 65 years of age. SDI values range from 1 to 100, with higher values reflecting greater deprivation. Additional details on SDI and relevant data can be found at https://www.graham-center.org/maps-data-tools/social-deprivation-index.html (accessed on 17 January 2024).

#### 2.2.5. Average Daily Air Quality PM2.5 Concentration

The 2016 census tract-level projections of PM2.5 levels obtained from the EPA’s Downscaler model [35] were used in our study. These data are provided by the National Environmental Public Health Tracking Network of the Centers for Disease Control and Prevention, and can be accessed at https://data.cdc.gov/Environmental-Health-Toxicology/Daily-Census-Tract-Level-PM2-5-Concentrations-2016/7vu4-ngxx; accessed on 17 January 2024.

**Figure 2:**
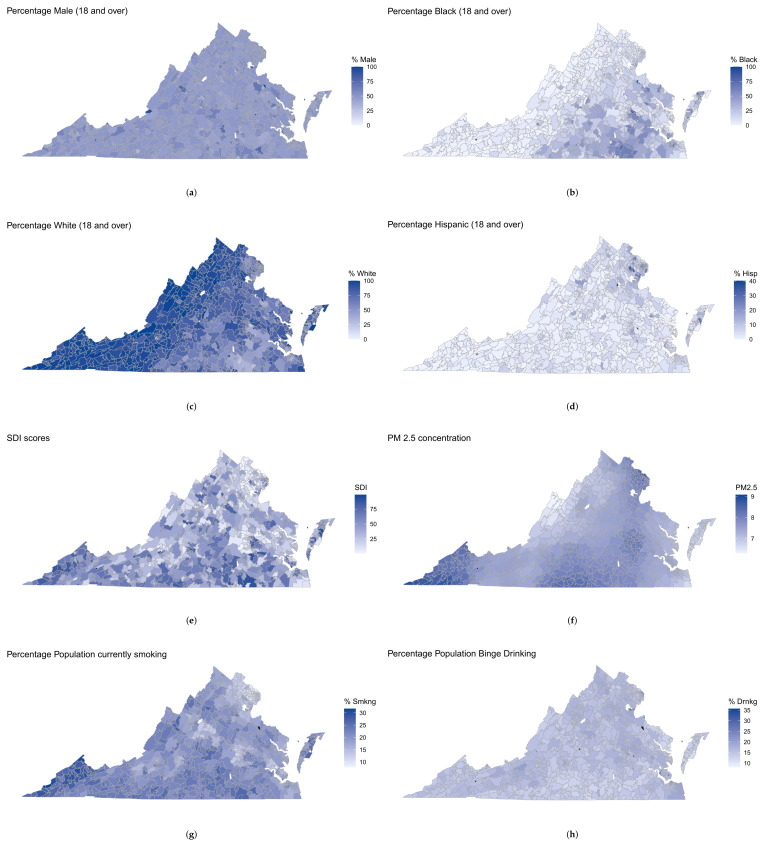
Zip code-level spatial maps of VA for (**a**) percentage of male population (18 and over), (**b**) percentage of Black population (18 and over), (**c**) percentage of White population (18 and over), (**d**) percentage of Hispanic population (18 and over), (**e**) Social Deprivation Index (SDI) scores, (**f**) PM2.5 concentration, (**g**) percentage of the population currently smoking, and (**h**) percentage of the population currently binge drinking. Zip codes with missing values for the percentage population currently smoking and binge drinking have been colored black.

Table 2 summarizes the characteristics of the above covariates. While the demographic variables, SDI scores, and PM2.5 concentration had no missing values, the percentages of population currently smoking, binge drinking, and suffering from obesity had missing values in seven zip codes (approximately 0.7% missing) and the percentage population below poverty had missing values in eleven zip codes (approximately 1.2% missing). Figure 2 shows the spatial variability of the covariates across VA. Zip codes with missing covariate values are colored black.

### 2.3. Spatial Statistical Model for LC Counts

Let $\left(y_{i},x_{i}\right)$ denote the data at the *i*th zip code (i=1,2,…,N), where $y_{i}$ is the LC count and the corresponding covariate vector is $x_{i}=\left(x_{i1},\ldots ,x_{ip}\right)$. A common approach to deal with count data is to model the random counts in the *i*th areal unit using a Poisson regression, as follows:

$$\begin{array}{cc} \; & y_{i}|\theta_{i}\overset{indep}{∼}Poisson\left(N_{i}\lambda_{i}\right),\;i=1,2,\ldots ,N\\ \; & log\left(\lambda_{i}\right)=x_{i}^{T}\beta +O_{i}+\theta_{i}, \end{array}$$

where β denotes the vector of regression parameters, $O_{i}=log\left(N_{i}\right)$ is the offset term, and $\theta_{i}$ is the spatial random effect included in model to account for any residual spatial autocorrelation in the data after accounting for the covariate effects. Let $\theta =(\theta_{1},\ldots ,\theta_{N})$ be a vector of spatial random effects, and let $N_{i}$ and $\lambda_{i}$ respectively denote the expected number and relative rate of LC at the *i*th zip code.

Under a Bayesian paradigm, a conditional autoregressive (CAR) prior is popularly assumed for θ; in particular, the *Besag* model [26], which models $\theta_{i}$ on the areal units i=1,…,N conditionally on neighbouring units, is often suggested. Two units are defined as neighbours when they share a common border. Thus, the conditional distribution for $\theta_{i}$ is

$$\theta_{i}|\theta_{-i},\tau ∼N\left(\frac{1}{m_{i}}\sum_{j∼i}\theta_{j},\frac{1}{m_{i}}\frac{1}{\tau }\right),$$

where $\theta_{-i}$ denotes the vector of spatial random effects excluding $\theta_{i}$, j∼i denotes that units *i* and *j* are neighbors, $m_{i}$ is the number of neighbors of the *i*th unit, and τ is the model precision. The joint distribution is provided by

$$\theta |\tau ∼N\left(0,\frac{1}{\tau }Q^{-1}\right),$$

where *Q* is the structure matrix provided by Q=M−ρW (with $M=diag(m_{1},\ldots ,m_{N})$), *W* is the adjacency matrix, defined as $W_{i,j}=1$ if i∼j and 0 otherwise, and ρ is the extent of spatial autocorrelation. An extension of the Besag model called the BYM model [26] additively combines an unstructured (nonspatial) random effect $\phi_{i}|\tau^{\prime }∼N\left(0,\tau^{\prime -1}\right)$.

### 2.4. Extension to the Negative Binomial Regression Model

As mentioned earlier, the observed LC counts exhibit overdispersion with $\mathrm{Var}\left(y\right)/\bar{y}=52.6>>2$ [36]. Because overdispersed data can lead to underestimated standard errors and inflated test statistics during estimation [37,38], switching from a Poisson to an NB regression model can be an effective solution. The NB distribution has an additional parameter to control data variability. The spatial regression model can now be expressed as

$$\begin{array}{cc} \; & y_{i}|\theta_{i}\overset{indep}{∼}\mathrm{NB}\left(r,\mu_{i}\right),\;i=1,2,\ldots ,N\\ \; & log\left(\mu_{i}\right)=x_{i}^{T}\beta +O_{i}+\theta_{i}, \end{array}$$

where r>0 is an additional scale parameter, $E\left(y_{i}|r,\theta_{i}\right)=\mu_{i}$, and $\mathrm{Var}\left(y_{i}|r,\theta_{i}\right)=\mu_{i}\left(1+\frac{\mu_{i}}{r}\right)$. The rest of the framework remains the same as defined in Section 2.3. The above parameterization of the NB model [39] implies that the variance is the mean multiplied by the positive factor $1+\mu_{i}/r$, and as such is greater than the mean, thereby modeling the overdispersion in the data.

When modeling epidemiological count data, it has been seen that Poisson models with spatial components often perform better than models accounting for overdispersion such as NB models [39]. Thus, we carried out fitting for both spatial models and compared their performances based on suitable model fitting criteria. In addition, nonspatial versions of the above models $\left(i.e.,\;\theta_{i}=0\;\mathrm{for}\;\mathrm{all}\;i=1,2,\ldots ,N\right)$ were fitted for comparison to their spatial counterparts.

### 2.5. Spatial Imputation of Missing Covariates

In quantifying the effect of factors related to LC, covariates pertinent to LC, such as prevalence of smoking and binge drinking, exhibit missing values; as a result, it was necessary to impute these missing covariates by accommodating the spatial framework to include them in our regression modeling. To this end, we implemented the imputation technique proposed by [30]. For simplicity, we consider the imputation of a single covariate with missing observations to analytically illustrate the method. However, during implementation this approach is extended to consider the imputation of missing values in several covariates using a multivariate CAR (MCAR) model [40].

Let D=(y,x) denote the complete dataset, where $x={\left(x_{obs},x_{mis}\right)}^{T}$ denote the complete set of values of a covariate. Here, $x_{obs}$ denotes the observed values of the covariate and $x_{mis}$ denotes the missing ones. We only impute missing values in covariates, as under the Bayesian setting models with missing response values can be easily fitted by computing the corresponding posterior predictive distribution [41]. Now, a latent effect $z=(z_{obs},z_{mis})$ is defined as a Gaussian Markov Random Field (GMRF, [31]) with a mean $\mu^{\prime }$ and precision matrix $Q^{\prime }$. Because the covariate is assumed to be spatially correlated, a proper CAR specification is used; thus, we have $\mu^{\prime }=\alpha$ and $Q^{\prime }=\tau_{c}\left(I-\rho W\right)$, where α is the intercept of the linear predictor, ρ is a spatial autocorrelation parameter, and *W* is the spatial adjacency matrix. Scaling *W* by its largest eigenvalue, such that ρ takes a value in (0,1), is suggested. After rewriting *W* as a block matrix with four submatrices according to the missing and observed values, then integrating the imputation model within a much larger model, the conditional distribution of $x_{mis}|x_{obs}$ is provided by

$$x_{mis}|x_{obs}∼N\left(\alpha_{mis}-{\left(I_{mis}-\rho W_{mis,mis}\right)}^{-1}\left(-\rho W_{mis,obs}\right)\left(z_{obs}-\alpha_{obs}\right),\tau_{c}{\left(I_{mis}-\rho W_{mis,mis}\right)}^{-1}\right),$$

### 2.6. Model Fitting and Model Comparison Using R-INLA

INLA [28,42] is a method for approximate Bayesian inference that allows for efficient estimation by relying on latent GMRFs. It is an established alternative to other simulation-based methods such as MCMC [43,44], and is preferred because of its computational efficiency, stability, and ease of use via the R-INLA package. For a gentle introduction to INLA, readers may refer to [41], with the online version available at https://becarioprecario.bitbucket.io/inla-gitbook/; accessed on 17 January 2024. The Besag model can be implemented as besagproper (the proper version of Besag’s spatial model), besag (Besag’s spatial model with improper prior), bym (the BYM model) [41], or generic0 in the inla function [45]. In addition, the popular Leroux CAR model [27], which offers a precision matrix that is a convex combination of an identity matrix (representing i.i.d. random effect) along with the precision of an intrinsic CAR, can be fitted using the generic1 model [46,47]. The latent effects can be incorporated as additive effects in the model formula.

However, the R-INLA algorithm does not accommodate ready handling of missing (covariate) values. When R-INLA encounters missing values in the covariates (denoted by NA in R), they are replaced by zeros, ensuring that the missing covariate does not affect the linear prediction. However, this results in biased estimates of the regression coefficients, as described in the R-INLA list of frequently asked questions (FAQ) on the package website. Missing values in the response variable are naturally imputed using the posterior predictive distribution. To circumvent this, we performed simultaneous (spatial) imputation of all missing covariates using latent GMRFs, as described above. When the imputed latent effect is included in the model formula, it becomes part of the joint latent effect and is incorporated into the Bayesian model, meaning that a full Bayesian approach can be employed to estimate all the model parameters (refer to [30] for full computational details, including the priors used in the latent effect). We used the MIINLA package in R (available from the Github repository https://github.com/becarioprecario/MIINLA; accessed on 17 January 2024) to implement the latent effects required to impute the missing covariate values. A simple CAR spatial latent effect was added to the model formula to capture the spatial random effects in the Poisson/NB regression model at the zip code level.

In the nonspatial versions of the implemented models ($\theta_{i}=0$ for all i=1,2,…,N in the models in Section 2.3 and Section 2.4), covariate imputations were made by linear regression [30] onto the set of fully observed covariates *X*. Let *X* be partitioned as $\left(\begin{array}{c} X_{obs}\\ X_{mis} \end{array}\right)$ to match the structure of $x={\left(x_{obs},x_{mis}\right)}^{T}$. Under this setting, the conditional distribution of the imputation model is provided by

$$x_{mis}|x_{obs}∼N\left(X_{mis}\beta ,\tau_{c}I_{mis}\right),$$

where $I_{mis}$ is the identity matrix and its dimensions depend on the number of missing values in *z*. For these models, the spatial neighborhood structure is ignored while imputing the covariates as well as during regression model fitting.

The fitted models (with and without covariate imputations) were evaluated using the Deviance Information Criteria (DIC) and the Watanabe–Akaike information criterion (WAIC; see [48]). For the sake of completeness, the DIC and WAIC are defined as follows:

$$\begin{array}{cc} DIC & =-2\;\log\;p\left(y|\hat{\theta }\right)+2p_{DIC}\\ WAIC & =-2\;\mathrm{lppd}+2p_{WAIC}, \end{array}$$

where $-2\;\log\;p(y|\hat{\theta })$ is the estimated deviance, $p_{DIC}$ and $p_{WAIC}$ denote the effective numbers of parameters when calculating DIC and WAIC, respectively, and ‘lppd’ denotes the log predictive pointwise density. The predictive performance of the fitted models was additionally measured by the Root Mean Squared Error (RMSE) and the Relative Squared Error (RSE) while using only the observed LC counts and their corresponding predicted values. The associated reproducible code (and vignette) for ready implementation of our methodology has been made available at https://github.com/indranil09/VA_LungCancer_INLA; accessed on 17 January 2024.

## 3. Results

In this section, we begin by providing model comparisons in Section 3.1, followed by a summary in Section 3.2 of our study findings utilizing the model with the best fit.

### 3.1. Model Comparison

Table 3 compares all fitted models based on popular model fitting and predictive performance criteria, described in Section 2.6. As suggested in [49], differences in DIC of more than 10 units constitutes an important difference, and can be considered as evidence to rule out the model with the higher DIC. Differences between 3–10 can be considered substantial. From the table, it can be concluded that the spatial Poisson GLM model, with missing covariates imputed using a multivariate CAR assumption, produces the best fit to our data. Because the Poisson model does not allow for overdispersion, this implies that the uncertainty due to overdispersion present in the data is adequately captured by the spatial random effects. In addition, the spatial Poisson model with no imputation is a close second, with comparable DIC, WAIC, RMSE, and RSE to the MCAR-imputed spatial Poisson model. This can be attributed to a small proportion of missing values in the covariates, while the gap between the performance of the two models is likely to increase as the percentage of missing values increases. Overall, the imputation models demonstrate better performance or, at the very least achieve comparable results compared to their non-imputation counterparts; the sole exception to this trend is observed for the spatially imputed NB model, which exhibits higher values for DIC, WAIC, RMSE, and RSE in comparison to its non-imputed counterpart. On the contrary, among the nonspatial models the NB model demonstrates substantial improvement over the Poisson model in terms of model fitting. The additional overdispersion parameter in the NB is now required to capture the data overdispersion. Additionally, the no-imputation spatial NB GLM performs best among all fitted NB models. The performances of the nonspatial imputed and non-imputed NB models are comparable due to low percentage of missing values and the omission of spatial random effects in the models.

### 3.2. Findings

The rest of this subsection summarizes the findings from the best-fitting model, i.e., the spatial Poisson regression model with multivariate CAR covariate imputation. These results are tabulated in Table 4, which shows the estimated posterior means, posterior standard deviations (SD), and 95% credible intervals of the regression parameters fitted using INLA. In the subsequent interpretations of a specific covariate, it is assumed that all other covariates were held as fixed. We observe that zip codes with a higher SDI score have a higher number of LC cases. This finding corroborates with previous studies [50], which concluded that LC is more prevalent in regions with lower income and education levels and among those living in more socioeconomically under-resourced regions. Surprisingly, zip codes with higher PM2.5 concentration reported lower LC prevalence. In terms of demographics, we observe that zip codes with higher Black and White populations reported higher LC counts, whereas zip codes with higher Hispanic population reported lower LC counts. These findings are consistent with recent studies conducted by the American Cancer Society’s Cancer Facts and Figures for Latino People (see, https://www.cancer.org/content/dam/cancer-org/research/cancer-facts-and-statistics/cancer-facts-and-figures-for-hispanics-and-latinos/hispanic-latino-2021-2023-cancer-facts-and-figures.pdf; accessed on 17 January 2024), which showed that LC prevalence rates among Hispanic men and women are about half those of non-Hispanic whites. This has been attributed to lower cigarette smoking prevalence among the Hispanic community. Our analysis further reveals that zip codes with higher male population (compared to females) report lower LC counts, suggesting that LC is more prevalent among women in VA compared to men. Similar conclusions have been drawn from nationwide studies performed during a contemporary study period [51,52]. Moreover, zip codes with a higher proportion of elderly individuals (≥65 years) reported higher LC counts, which is in tune with the trend across the United States [53]. Zip codes with a higher smoking index reported higher LC counts, while zip codes with a higher binge drinking index had fewer LC cases. The obesity index was not found to be statistically significant. Finally, zip codes with higher poverty levels reported lower LC prevalence. This is counter-intuitive, as people with lower annual family income and socioeconomic status usually experience higher LC prevalence [54]. One potential factor contributing to this might be the financial constraints faced by individuals residing in high-poverty zip codes, which appear as an impediment to LC screening through eligible health insurance plans. Based on data collected between May to July 2022 by the American Lung Association, it is notable that VA Medicaid fee-for-service programs extend coverage for low-dose computed tomography-based lung cancer screenings to individuals deemed at high risk for lung cancer without any prior authorization and with no co-pay (see, https://vamedicaid.dmas.virginia.gov/vamed/download-pdf-bulletin/18381; accessed on 17 January 2024). However, VA follows the updated 2021 USPSTF (United States Preventive Services Task Force) guidelines regarding patient eligibility, which stipulate individuals between ages 55 and 80 years with a smoking history of 20 packs per year and who are either a current smoker or have quit smoking within the last 15 years [55]. This counter-intuitive trend can be further attributed to the fact that the apparent pattern has already been addressed by the inclusion of the Social Deprivation Index (SDI) variable in the model.

The competing models provide very similar parameter estimates and interpretations of the relationship between the LC counts and the covariates, except for the non-spatial Poisson model with linear regression imputation, which additionally shows a positive effect of obesity on LC counts.

Additionally, when comparing the estimated overdispersion parameters between the spatial and nonspatial NB imputation models, we observe that the NB overdispersion parameter under the nonspatial setting is much larger than that under the spatial setting. This implies that part of the data variability in the overdispersed LC counts is explained by the spatial random effects, rather than the *r* parameter alone. This tendency of spatial models is well documented [39]. Similar trends were observed between the NB spatial and nonspatial model fits with no covariate imputation.

**Figure 3:**
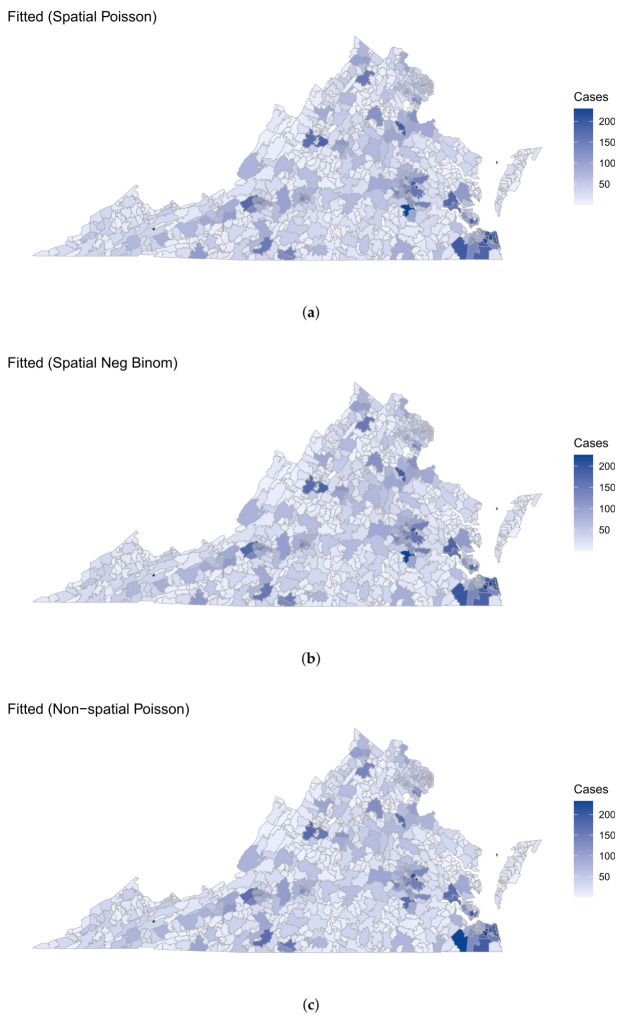

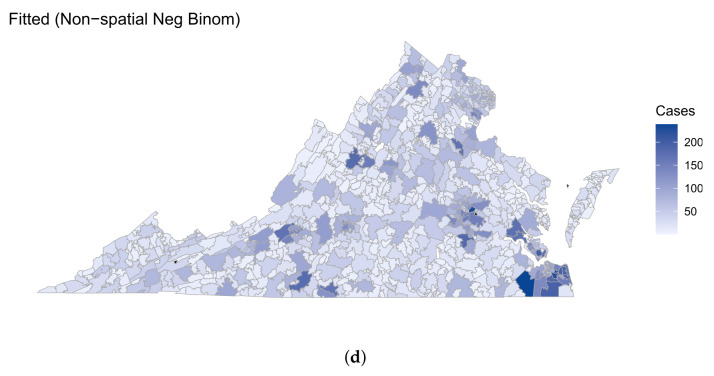
Zip code-level spatial maps of fitted LC counts obtained from (**a**) the spatial Poisson model, (**b**) the spatial NB model, (**c**) the nonspatial Poisson model, and (**d**) the nonspatial NB model. For all four models, missing covariates were simultaneously imputed using the multivariate CAR model (for spatial models) and linear regression (for nonspatial models).

Figure 3 shows the fitted LC counts in VA using the spatial and nonspatial Poisson and NB GLM models after imputing the missing covariates. Both spatial imputation models are able to correctly identify zip codes in Petersburg (23083) and VA Beach (23452 and 23464) as the top three zip codes with highest LC prevalence. On the other hand, the nonspatial imputation models point out zip code 23434 in Suffolk as the zip code with highest LC prevalence, followed by Richmond and VA Beach. While all four models are able to capture the spatial distribution of LC counts across VA, the nonspatial models do so based solely on the spatial variation attributed to the covariates, and as such tend to underestimate the higher counts in the data while overestimating the lower counts.

## 4. Conclusions

The examination of LC cases in VA is of paramount importance due to its far-reaching implications for public health. In this comprehensive study, we have engaged in a thorough analysis to determine those demographic, environmental, and socioeconomic factors which influence the prevalence of LC at the zip code level in VA. LC count data are usually overdispersed, with relevant covariates often experiencing missing values. For this reason, we utilized Poisson and NB spatial regression models to predict LC counts while accounting for overdispersion in the response. To handle missing values, we performed simultaneous spatial imputation of missing covariates by treating them as latent GMRFs within a Bayesian hierarchical modeling framework powered by INLA. The findings from this study provide invaluable insights into the multifaceted determinants of LC, making a substantive contribution to the field of public health. Our study effectively pinpoints zip codes characterized by the highest prevalence of LC. A central goal of spatial data analysis is hotspot detection, as information regarding specific zip codes can be instrumental in guiding and prioritizing tailored interventions at the zipcode level during policy implementations aimed at mitigating the impact of this pervasive health concern in the state of VA with an eye towards resource allocation. Although the conclusions drawn from our findings are specifically tailored to aggregated LC counts at the zip code level in VA spanning the years 2014 to 2018, the statistical methodology and framework we have proposed is generalizable to any disease incidence or prevalence data exhibiting features of overdispersion, spatial variability, and covariate missingness, and the accompanying implementation code has been provided on GitHub.

The current study is not aloof from limitations, which we discuss below. While our study incorporates smoking indices as a covariate, it is crucial to note that the observational nature of the study restricts us from drawing definitive conclusions regarding any causal relationship between smoking and LC. In order to assess smoking–lung cancer causality, it would be necessary to use a spatial causal inferential framework while mitigating the challenges of overdispersion and missing covariate information (such as a spatial–causal framework in the presence of unmeasured confounders [56], which can arise due completely missing/unobserved/mismeasured covariates or an incorrect functional form in the outcome model. Moreover, smoking indices do not consider the situation in which measured covariates have missing information. Thus, we could potentially look to incorporate missing covariate imputation into a typical spatial–causal framework in order to work with the data at hand. In addition, exploring spatial causal inference with multiple risk factors contributing to LC prevalence is possible, and would enable evaluation of the effectiveness of interventions and policies targeted at reducing the burden of LC within specific zip codes. By understanding spatial causality, policymakers can allocate resources more efficiently, implement targeted prevention strategies, and tailor healthcare interventions at the zip code level to address the specific needs of high-risk populations in particular zip codes. Furthermore, the current exploration is cross-sectional, combining LC counts across 5 years, under the assumption that individuals diagnosed with LC reside within the same zip code throughout this 5-year period. This approach provides a spatiotemporal perspective on cancer prevalence rather than a longitudinal one. Consequently, our analysis does not consider policies implemented during the study period, precluding the identification of the effectiveness of specific policies in mitigating cancer prevalence. Certainly, a more interesting approach (subject to availability of data) would be to incorporate a spatiotemporal model (justifiably nontrivial when factoring in the other covariate complications) over an extended time frame, which would permit variations in zip code populations and thereby capture the dynamics of individual migration across different regions over time. All of these represent potential avenues for future research and will be considered elsewhere.

## Figures and Tables

**Table 1:** Top 15 zip codes in VA which recorded the highest number of LC cases over the five-year period 2014–2018.

Zip Code	City	County	LC Counts
23803	Petersburg	Petersburg (City)	234
23452	VA Beach	VA Beach (City)	230
23464	VA Beach	VA Beach (City)	218
23462	VA Beach	VA Beach (City)	202
23455	VA Beach	VA Beach (City)	199
23454	VA Beach	VA Beach (City)	197
23434	Suffolk	Suffolk (City)	196
23320	Chesapeake	Cheasapeake (City)	194
22407	Fredericksburg	Spotsylavnia	194
23451	VA Beach	VA Beach (City)	193
23223	Richmond	Richmond (City)	189
22980	Waynesboro	Waynesboro (City)	188
23322	Chesapeake	Cheasapeake (City)	181
24153	Salem	Salem (City)	180
23666	Hampton	Hampton (City)	177

**Table 2:** Covariate summaries from the VA LC data with subjects ≥18 years in age. Means and standard deviations were calculated over n = 890 zip codes for covariates with no missing values. For covariates with missing values, means and standard deviations were calculated over the observed zip codes.

Variables	Mean (SD)
Gender (percent)	
Female	50.94 (8.80)
Male	49.06 (8.80)
Race (percent)	
Black	16.38 (18.84)
White	77.95 (20.24)
Other (Asian, NHPI, two or more races, etc.)	5.67 (7.84)
Ethnicity (percent)	
Hispanic or Latino	4.05 (5.83)
Not Hispanic or Latino	95.95 (5.83)
Percent population with age ≥65	24.43 (13.16)
Percent population currently smoking	19.87 (4.40)
Percent population binge drinking	15.99 (2.80)
Percent population obese	33.92 (5.40)
Percent population below poverty	11.46 (9.80)
Social Deprivation Index (SDI)	38.09 (24.95)
Daily Air Quality PM2.5 Concentration	7.58 (0.48)

**Table 3:** Comparison of the competing model fits to the VA LC data using various model fitting (DIC, WAIC) and prediction accuracy (RMSE, RSE) criteria.

Model	DIC	WAIC	RMSE	RSE
*MCAR Imputation model*				
Spatial Poisson GLM	5007.13	5010.27	3.55	0.0072
Spatial NB GLM	5062.60	5057.44	4.06	0.0095
*Linear regression Imputation model*				
Non-spatial Poisson GLM	5446.90	5465.77	8.94	0.046
Non-spatial NB GLM	5205.08	5207.39	9.56	0.052
*No Imputation model*				
Spatial Poisson GLM	5011.08	5012.52	3.56	0.0073
Spatial NB GLM	5041.98	5040.88	3.85	0.0085
Non-spatial Poisson GLM	5446.34	5464.33	8.94	0.046
Non-spatial NB GLM	5207.47	5210.06	9.55	0.052

**Table 4:** Estimated posterior means, posterior standard deviations, and 95% credible intervals for the regression parameters obtained from fitting the Poisson spatial regression model to the VA LC data. Here, missing covariate values have been imputed simultaneously during model fitting using a multivariate spatial model.

Covariates	Posterior Mean	Posterior SD	95% Credible Interval
SDI score	0.050	0.014	(0.021, 0.076)
PM2.5	−0.089	0.015	(−0.118, −0.059)
Race (w.r.t. Others)			
% black	0.212	0.046	(0.122, 0.301)
% white	0.255	0.046	(0.165, 0.345)
Ethnicity (w.r.t. non-hispanic)			
% hispanic	−0.039	0.015	(−0.068, −0.009)
Gender (w.r.t. female)			
% male	−0.096	0.020	(−0.137, −0.056)
Age (w.r.t. < 65 years)			
% over 65 years	0.211	0.022	(0.167, 0.254)
Binge drinking idx	−0.103	0.023	(−0.150, −0.058)
Smoking idx	0.136	0.030	(0.075, 0.192)
Obesity idx	0.046	0.030	(−0.011, 0.108)
Poverty idx	−0.083	0.026	(−0.135, −0.031)

## Data Availability

The dataset and code used for this project can be found at: https://github.com/indranil09/VA_LungCancer_INLA; accessed on 17 January 2024.
